# Antigenic epitope targets of rhesus macaques self-curing from *Schistosoma mansoni* infection

**DOI:** 10.3389/fimmu.2023.1269336

**Published:** 2024-02-23

**Authors:** Gillian M. Vance, Mariana I. Khouri, Almiro Pires da Silva Neto, Sally James, Luciana C. C. Leite, Leonardo Paiva Farias, R. Alan Wilson

**Affiliations:** ^1^ Department of Biology, University of York, York, United Kingdom; ^2^ Laboratório de Inflamação e Biomarcadores, Instituto Gonçalo Moniz, Fundação Oswaldo Cruz, Salvador, BA, Brazil; ^3^ Laboratório de Desenvolvimento de Vacinas, Instituto Butantan, São Paulo, SP, Brazil; ^4^ Biomedical Research Institute, University of York, York, United Kingdom

**Keywords:** epitope mapping, tegument proteins, alimentary tract proteins, antigenic targets, esophageal glands, peptide array

## Abstract

The self-cure of rhesus macaques from a schistosome infection and their subsequent strong immunity to a cercarial challenge should provide novel insights into the way these parasites can be eliminated by immunological attack. High-density arrays comprising overlapping 15-mer peptides from target proteins printed on glass slides can be used to screen sera from host species to determine antibody reactivity at the single epitope level. Careful selection of proteins, based on compositional studies, is crucial to encompass only those exposed on or secreted from the intra-mammalian stages and is intended to focus the analysis solely on targets mediating protection. We report the results of this approach using two pools of sera from hi- and lo-responder macaques undergoing self-cure, to screen arrays comprising tegument, esophageal gland, and gastrodermis proteins. We show that, overall, the target epitopes are the same in both groups, but the intensity of response is twice as strong in the high responders. In addition, apart from Sm25, tegument proteins elicit much weaker responses than those originating in the alimentary tract, as was apparent in IFNγR KO mice. We also highlight the most reactive epitopes in key proteins. Armed with this knowledge, we intend to use multi-epitope constructs in vaccination experiments, which seek to emulate the self-cure process in experimental animals and potentially in humans.

## 1 Introduction

Despite the introduction of Praziquantel 45 years ago as a safe and effective drug (1) and, more recently, its use in mass chemotherapy, schistosomiasis stubbornly remains a public health problem in many countries where the disease is endemic. In 2019, the World Health Organization estimated in that at least 236.6 million people required preventive treatment for schistosomiasis (https://www.who.int/news-room/fact-sheets/detail/schistosomiasis), and given the increasing sensitivity of diagnostic techniques, that figure could be significantly higher (2, 3). Inevitably, this has led to calls for the development of vaccines as additional components in the toolbox of control measures (4). However, progress towards an effective schistosome vaccine has been slow, not least because of the difficulty in demonstrating specific acquired immunity following infection. The animal and human trials with single recombinant antigens have at best yielded only partial protection of uncertain duration (5, 6).

Nevertheless, work with animal models does indicate that substantial levels of protection can be induced. The radiation-attenuated (RA) cercarial vaccine delivered to both rodents and non-human primates is the most intensively researched (7, 8). Multiple exposures are required to achieve high levels of protection, the underlying immunological mechanisms are complex, and in the baboon, host immunity declines in parallel with antibody titer in the months after the last vaccination (8). The RA vaccine, with its shelf-life of hours, is best viewed as an experimental model to dissect the mechanism of protection, rather than a practical proposition. A second model, the rhesus macaque, was favored in early studies of protective immunity (43 papers cited on PubMed in the 1960s) before being replaced by cheaper rodent models. The principal attraction was the animal’s ability to clear an established infection in a self-cure process, after which it was resistant to a cercarial challenge (9, 10). Given the slow progress with recombinant antigens, the model has been revisited in short-term (18–22 weeks) experiments with both *S. mansoni* (11) and *S. japonicum* (12) in the hope that it would provide pointers to the immune mechanisms, which can eliminate both established and challenge populations. Most recently, this work has been extended to a single large cohort of rhesus macaques given a primary infection followed by a cercarial challenge at 42 weeks, with protection determined at 60 weeks (13). In summary, the self-cure process begins approximately 10 weeks post-exposure but proceeds at different rates in individual animals. The animals that self-cured the most rapidly were the ones that showed the greatest degree of protection, some with virtually sterile immunity; no challenge worms achieved egg excretion, even in the slowest responders. Although the above models demonstrate acquired protection against a schistosome infection, it is no simple task to identify the antigens mediating that process in a macroscopic pathogen with a genome comprising ~12,000 protein coding genes, expressed in multiple organ systems. For this reason, array technologies have been developed for screening of sera from protected animals to assess their immune reactivity. Arrays comprising fractionated glycans have been used to identify the reactive epitopes in sera from rhesus macaques self-curing from *S. japonicum* (14) and baboons after multiple exposures to the RA *S. mansoni* vaccine (15). It remains unclear if these glycan antigens are involved in the protective response or provide a smokescreen to divert attention away from protective epitopes (16).

The rhesus macaque serum taken at weeks 12 and 20 has also been used to screen a large-scale array comprising *in vitro* translated proteins of *S. mansoni* (45) and *S. japonicum* (172) printed onto glass slides (17). Eight proteins were detected by the 22-week rhesus serum pool, but the only plausible candidate was an extracellular superoxide dismutase of unknown localization. The reactivity of target proteins can now be evaluated at the level of individual epitopes by printing of overlapping 15mer peptides onto glass slides for screening with immune sera. The technique was first applied to three such arrays encompassing 33 esophageal proteins from *S. japonicum* screened with serum from self-curing macaques, infected rabbits, and mice (18). While some reactivities were common to the three hosts (e.g., MEGs 4.1, 4.2, 11, 12, and an aspartyl protease), those to MEG-8.1 and 8.2 were largely confined to the macaques. Expanding on this epitope mapping approach, we have screened four peptide arrays comprising 55 secreted or exposed proteins from the alimentary tract and tegument of *S. mansoni with* sera from C57Bl/6 and IFNγR KO mice after multiple exposures to the RA vaccine. A list of priority peptides from 44 of the proteins was obtained for further investigation in multiepitope vaccine constructs and as targets of monoclonal antibodies (19). We now describe the reactivity of two pools of serum from rhesus macaques self-curing from *S. mansoni* (11) against the same four peptide arrays. We show that titer, not target, appears to determine the rate of self-cure, and we highlight the principal reactive epitopes of alimentary tract and tegument proteins for inclusion in a multi-epitope construct for vaccine experiments.

## 2 Methods

### 2.1 Source of self-curing sera and ethics statement

The high- and low-responder pools of rhesus sera used in the array screen came from the schistosomiasis serum archive at the University of York, acquired in the previously published study of self-cure undertaken at the Biomedical Primate Research Centre (BPRC), Rijswijk, The Netherlands (11). The experimental protocol was approved by the Institutional Animal Care and Use Committee at BPRC and the Biology Department Ethics Committee, University of York. The animals were exposed to 1,000 *S. mansoni* cercariae via the shaved abdominal skin for 30 min and the serum obtained by intravenous sampling at perfusion (week 18). The hi-responder pool combined serum from rhesus macaque R1 (12) and R6 (31) and the lo-responder pool from R2 (708) and R5 (249); numbers in parentheses are worms recovered. We also made computational comparisons with two of our previously published array studies: 1. IFNγR KO mice, which were capable of conferring ~50% passive protection on naive recipient mice (19) and 2. rhesus macaques self-curing from *S. japonicum* infection (18).

### 2.2 Array design and screening

The four previously described array designs (19) consisting of overlapping 15mer peptides, with a one, two, or three amino acid offset, were printed by PEPperPRINT (Heidelberg, Germany; https://www.pepperprint.com). The array slides were screened exactly as previously described (18, 19). These are the same arrays that were first reacted with mouse primary serum samples, with binding detected by Cy3-labeled goat anti-mouse IgG (19). A total of 55 alimentary tract and tegument proteins, exposed at or secreted from the intra-mammalian stages, were investigated, primarily selected by the presence of a signal peptide (which was excised from the sequence printed). Rhesus primary antibodies were applied at a 1:200 dilution for alimentary tract proteins and 1:100 for the tegument. Their binding was detected using Cy5-labeled goat anti-human IgG (H+L), pre-adsorbed using bovine, chicken, horse, mouse, pig, rabbit, and rat immunosorbents to remove cross-reactive antibodies (Abcam #97172), at 1:300 dilution. The two-color detection allowed the direct comparison of previously identified murine responses with the rhesus macaque reactivities. Detection specificity was confirmed by a pre-stain of each array with Cy5-labeled secondary antibody and a preliminary scan. Blocking, secondary antibody, and control antibody solutions were each incubated at room temperature for 30 min; primary antibody solutions were incubated overnight at 4°C. Arrays were scanned at 5 µm resolution using an Agilent Array scanner with High-Resolution SureScan Technology (Agilent Technologies LDA UK Limited, Stockport, Cheshire; model G2565CA). The instrument has a dynamic range > four orders of magnitude; by optimizing antibody dilutions, the arrays were never saturated, while weaker reactivities were still captured. A screengrab of the Agilent image was taken for orientation and editing purposes.

### 2.3 Data analysis

The Agilent.tif file output for each array was analyzed using the PepSlide^®^ Analyzer (PSA) software as previously described (18). Heatmaps were then made from the cell scores for each array to facilitate visual interpretations. As the same aliquot of Cy5-Goat anti-human detection reagent was used throughout, the mean PSA scores for each position on the array allow comparisons of the intensity of reactive regions between individual samples. PSA scores were color-coded on a linear scale using the Conditional Formatting function in Excel to highlight reactive regions and facilitate comparisons between samples. An aggregate score for each reactive region was determined by summing adjacent peptide means above a predetermined threshold, down the array. In turn, these aggregates were combined to give a reactivity score for each of the 55 proteins under investigation. Additionally, we generated a reactivity-normalized score by dividing this last score by the total number of peptides for each protein. The sequence identity between the epitopes from *S. mansoni* and *S. japonicum* was calculated using the biostrings R package (https://bioconductor.org/packages/Biostrings). The two corresponding epitopes were aligned using the “pairwise Alignment” function with the global alignment option.

## 3 Results

### 3.1 Overview: proteins from the alimentary tract are generally more reactive than those from the tegument

**Figure 1:**
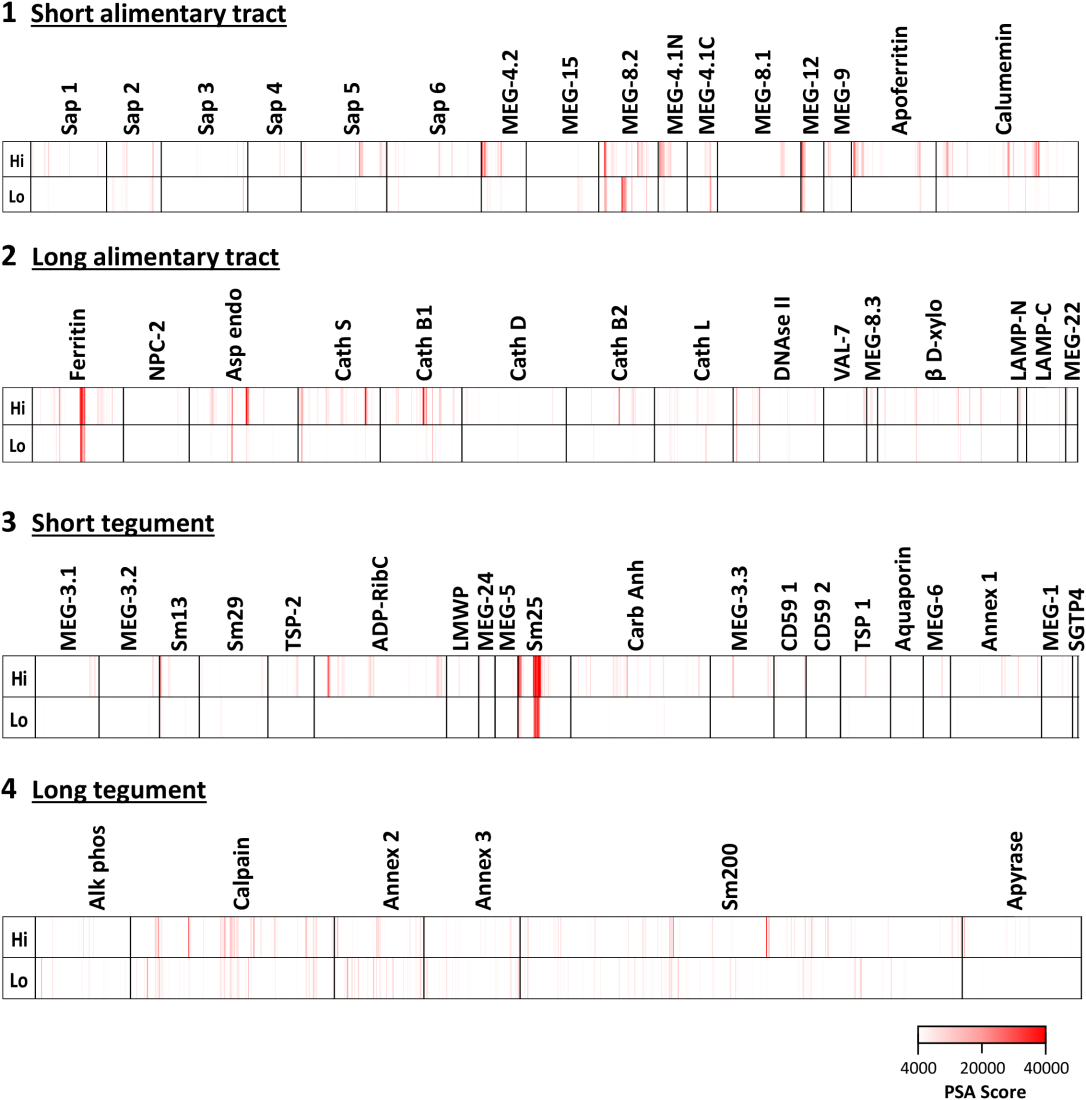
Heatmaps showing the reactivities of hi- and lo-responder rhesus macaque serum pools against the four peptide arrays: 1) short alimentary tract, 2) long alimentary tract, 3) short tegument surface, and 4) long tegument surface. The intense reactivity of tegument Sm25 is very evident.

The full data set obtained from screening the four arrays using high- and low-responder pools is provided in **Supplementary Table S1** and presented in **Figure 1** as a series of color-coded heatmaps to indicate the intensity of IgG binding against each 15mer peptide. The highest tegument array cell score was 44,974 units (Sm25) and for the alimentary tract, 55,163 units (ferritin heavy chain) (**Supplementary Table S1**). The number of neighboring reactive 15mer peptides ranged up to 20–23 (Sm25, calumenin), but was mostly smaller, in many cases likely representing a single epitope (**Supplementary Table S1**). Inspection of the heatmaps (**Figure 1**) reveals that, with one exception, the intensity of reactivity of the proteins printed on Array 1 (gastrodermal carrier proteins and esophageal secreted MEGs) and Array 2 (largely gastrodermal enzymes) showed the strongest reactivity. The striking exception is the short tegumental protein, Sm25 on Array 3, which shows the strongest overall visual signal. It is also apparent that the pool of hi-responder serum is more reactive against all the arrays, than the lo-responder pool, reacting weakly or not at all with the same targets. Only in MEG-8.2 does there appears to be a reactivity unique to the lo-responders.

### 3.2 Esophageal MEG and gastrodermal targets

**Figure 2:**
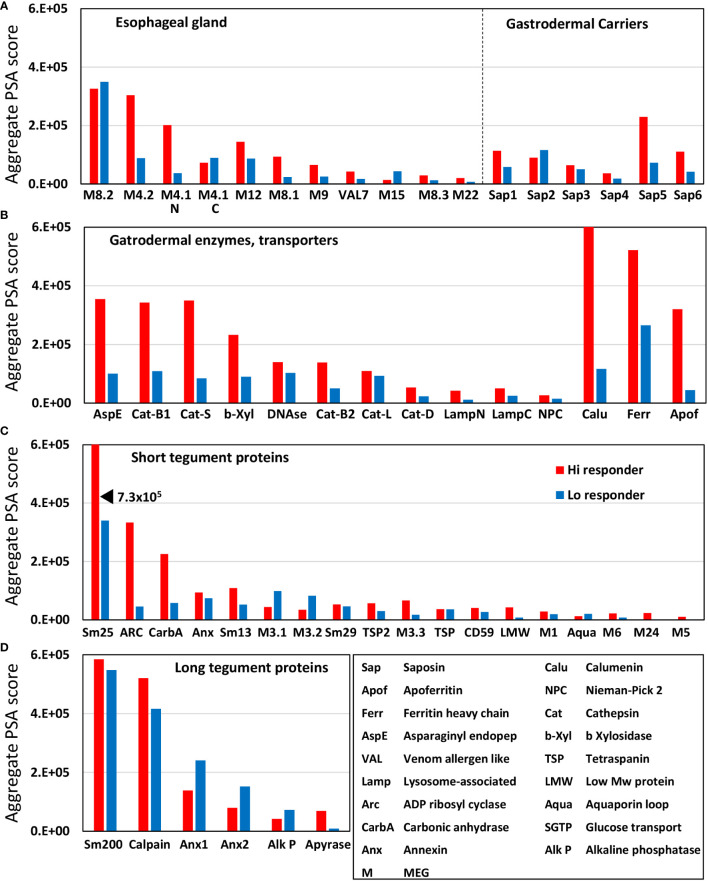
Bar chart summarizing the reactivity of all proteins on the four arrays, based on the data in **Supplementary Table S2**. The y-axis is the cumulative Agilent peptide score above zero for each protein, ignoring protein length. The proteins in each array have been rearranged along the x-axis according to functional group by reactivity. Three transporters with high reactivity on Array 2 were segregated on the basis of SchistoCyte predictions of their tissue localization. Note that the tegument arrays were hybridized with double the concentration of serum used for the alimentary tract arrays (1:100 versus 1:200).

The complexity of data presented in the heatmaps can be further reduced by summing the reactive regions within each protein (**Supplementary Table S2**) and plotting them in a bar chart (**Figure 2**). Viewed together, the heatmap (**Figure 1**) and bar chart (**Figure 2**) permit the proteins in each tissue of origin (**Figure 1**) to be graded by their overall reactivity. MEG-8.2 and MEG-4.2 were the most reactive esophageal secreted proteins followed by MEG-4.1 N and C termini and MEG-12. The MEG-8.1, MEG-9, VAL-7, and MEG-15 proteins reacted in descending order, while MEG-8.3 and MEG-22 were the weakest of all. However, four of these proteins reacted primarily at a single region: MEG-8.3 centrally, MEG-12 and MEG-22 at the extreme N-terminus, and VAL-7 at the C-terminus (**Figure 1**; **Supplementary Table S2**).

Among the gastrodermal secreted proteases, asparaginyl endopeptidase was the most reactive followed by cathepsins B1 and S (**Figure 2B**). Cathepsins B2 and L, plus beta xylosidase and DNAse, were moderately reactive over multiple regions of sequence. In contrast, Cathepsin D plus the N- and C-terminus of lysosomal-associated membrane protein, LAMP-1, were only weakly reactive. Among the lipid-transporting proteins secreted from the gastrodermis, only saposin-5 was moderately reactive, the others weakly so (**Figure 2A**), and the cholesterol-transporting Niemann-Pick 2 (NPC2) was barely detected (**Figure 2B**). In light of the single-cell sequencing results reported by Wendt et al. (2020), the strong reactivity of calcium binding calumenin and iron binding ferritin heavy chain may possibly originate in non-gastrodermal tissues.

### 3.3 Few tegument proteins show marked reactivity

Sm25 of unknown function was by far the most reactive tegument protein, more so in the hi- than lo-responder pool (**Figure 2C**). Among potential membranocalyx constituents, only Sm200 showed moderate reactivity (**Figure 2D**), but equally so in hi- and lo- responder pools. The vaccine candidate Sm-p80 calpain was similarly reactive with both pools (**Figure 2D**). The GPI-anchored tegument enzymes, ADP-ribosyl cyclase (Arc) and Carbonic anhydrase, on the outer leaflet of the plasma membrane, showed moderate reactivity with the hi-responder pool (**Figure 2C**). Finally, the three tegument annexins came next in rank order, two of them detected more strongly by the low responder pool. In contrast, GPI-anchored alkaline phosphatase and membrane-spanning apyrase (ATP-diphosphohydrolase), with a large extracellular domain, showed weak reactivity (**Figures 2C, D**). The N-terminus of Sm13 was weakly reactive with the hi-responder pool (**Figure 1**; **Supplementary Table S2**).

### 3.4 Normalizing by number of peptides printed allows ranking of protein reactivity

**Figure 3:**
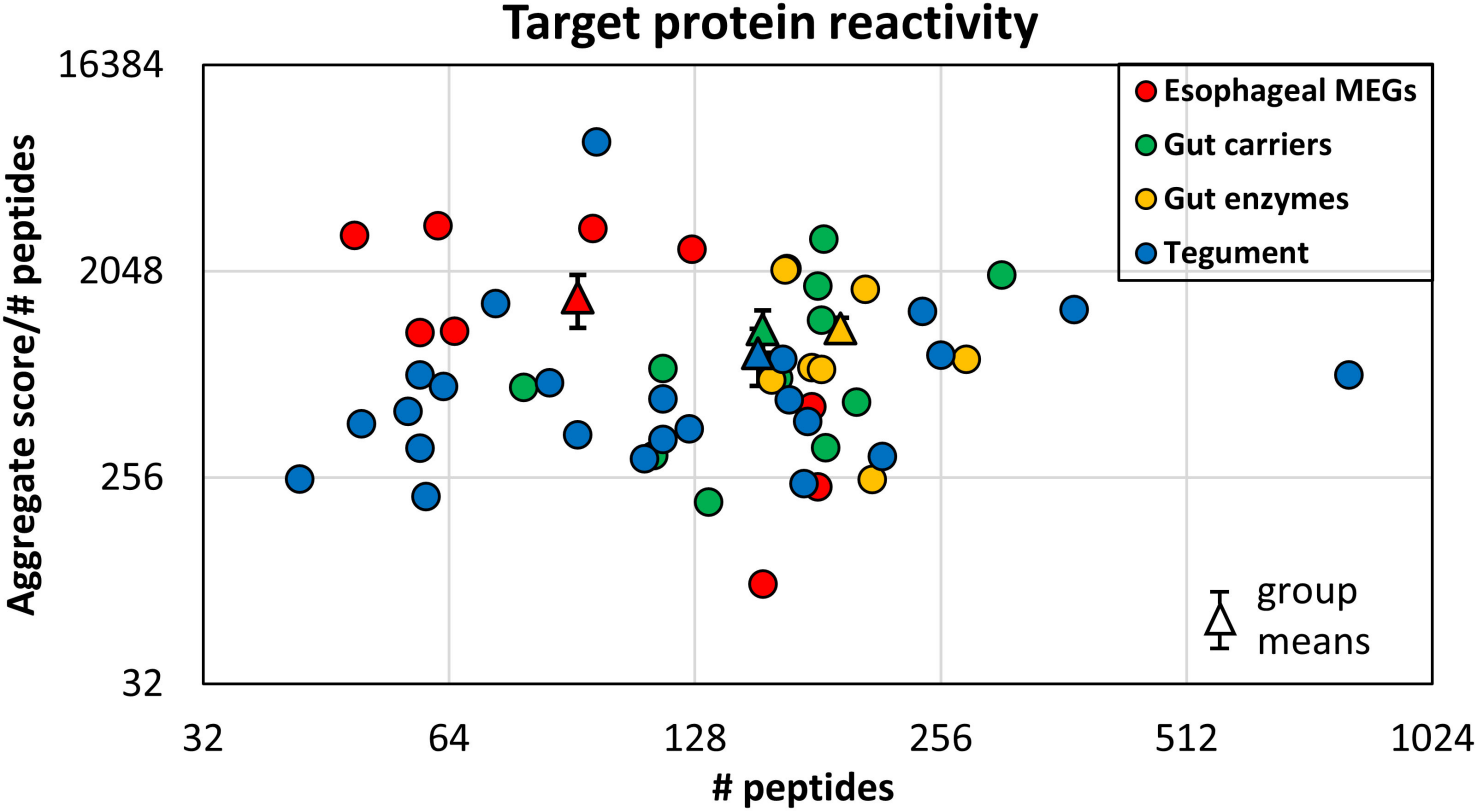
The mean reactivity of array proteins based on the data in **Supplementary Table S3**, normalized for number of peptides printed. This compensates for one, two, or three amino acid offsets. The proteins are color coded by function as esophageal MEGs, gastrodermal carriers, enzymes, and tegument. The differences in group reactivity were tested for significance using a t-test. Note the log x- and y-axis scales.

The proteins printed on the four arrays differ in size from 8 to 200 kDa, and the amino acid offsets of the 15mer peptides vary between one and three, to accommodate the larger ones. This divergence was normalized to create a reactivity index by dividing the mean score (**Supplementary Table S3**) by the total number of peptides printed for each protein. The esophageal MEGs plus VAL-7 are the smallest targets ( $\bar{x}$ 100 amino acids (AA). The 17 tegument proteins range between 44 and 1,630 AAs ( $\bar{x}$ 262, skewed by Sm200; median = 129). The gastrodermal carriers plus LAMP are somewhat larger ( $\bar{x}$ 166 AA), while, overall, the secreted hydrolases are the bulkiest targets ( $\bar{x}$ 414 AA). The indexes, sorted by tissue of origin and displayed as a scatter plot (**Figure 3**), segregate into four distinct but overlapping groups. The esophageal MEGs cluster at the top left ( $\bar{x}$ 1569 +/− S.E. 344). The gastrodermal carriers ( $\bar{x}$ 1143+/− S.E. 255) and enzymes ( $\bar{x}$ 1153 +/− S.E. 250) form a superimposed cluster in the center. Finally, the tegument proteins ( $\bar{x}$ 898 +/− S.E. 286) spread across the whole plot with the lowest score. It is notable that no group mean is statistically significantly different from any other (all p-values > 0.05, NS). However, there are two outliers, Sm25 highly reactive for the tegument and MEG-15 under-reactive for the esophageal gland. If these are removed before analysis, then the mean tegument score ( $\bar{x}$ 621 +/− S.E. 74) is significantly lower than the MEGs ( $\bar{x}$ 1717 +/− S.E. 331,***), carriers ( $\bar{x}$ as above,**) and enzymes ( $\bar{x}$ as above,**) of alimentary tract origin (**Supplementary Figure S1**).

### 3.5 Hi-responders react more strongly with array peptides than lo-responders

**Figure 4:**
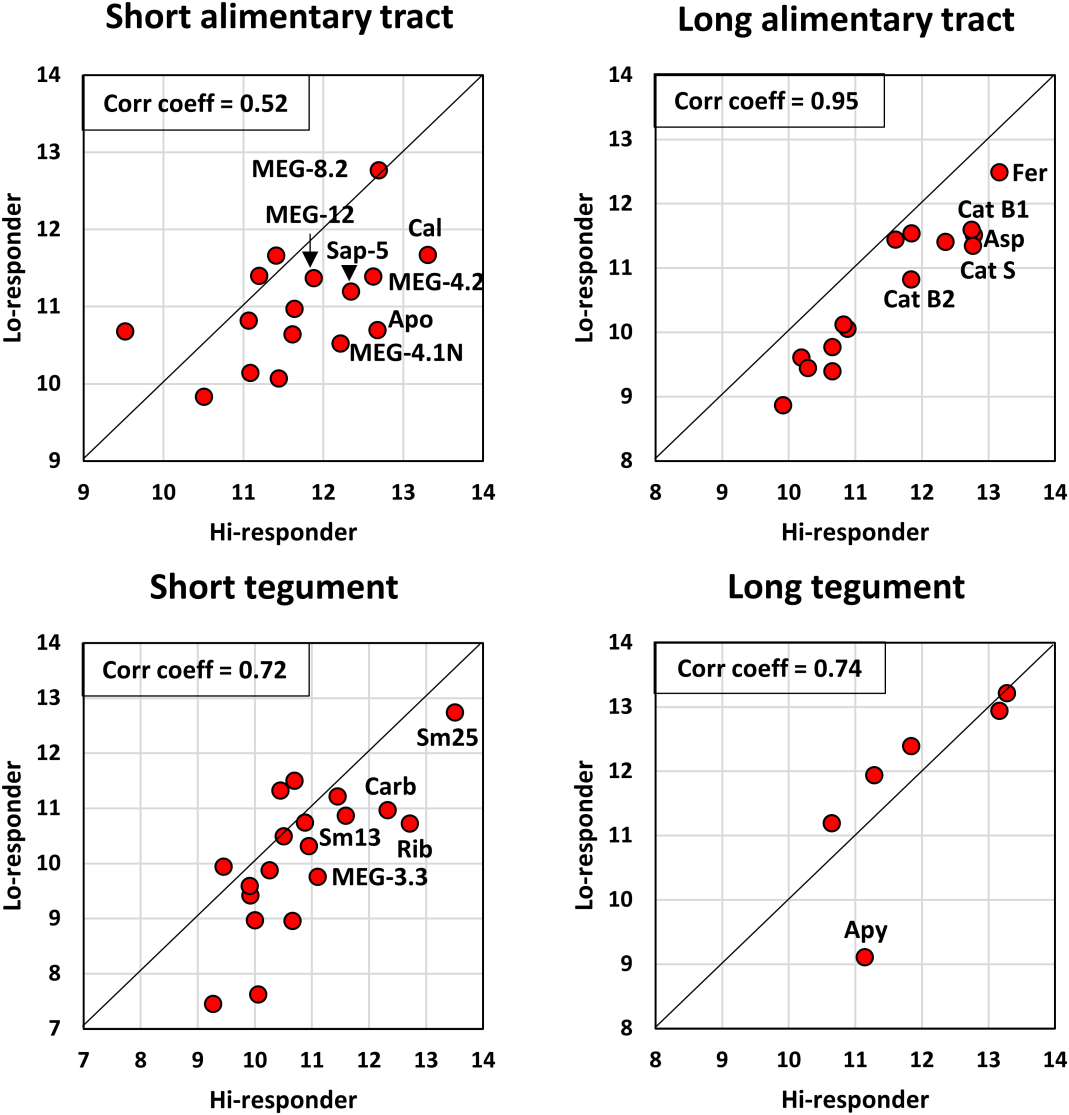
The reactivity of hi- and lo-responder pools compared for each array, based on the data in **Supplementary Table S2**. The linear relationship between the two variables was determined using Pearson’s correlation coefficient, r. The offset from the diagonal midline provides a visual indication of the extent to which each protein reacts more with the hi-responder pool.

It is clear from visual inspection of the bar charts (**Figure 2**) and the grand total aggregate scores for each array (**Supplementary Table S3**) that the hi-responder pool generally reacts more strongly with the 55 proteins printed on the arrays than the lo-responder pool. This is an important consideration when selecting target epitopes or proteins and can be visualized using an LN transform scatter plot of the aggregate scores for all proteins on the four arrays (**Figure 4**; **Supplementary Table S3**). The hi and lo scores for individual proteins on the long alimentary tract array 2 are strongly correlated (r = 0.95), but all hi-responder values are displaced from the median line by a factor of 2.4 times. The second highest correlation is between the six long tegument proteins (r = 0.74), but five of these lie along the median line, suggesting that responses to them are not a dominant component of self-cure. Only apyrase is an outlier, skewed towards the hi-responder pool, but the weakest of the six. The reactivity of short tegument proteins is moderately correlated (r = 0.72), twice as reactive with the hi-responder pool. Based on intensity and differential reactivity, Sm25, carbonic anhydrase, Sm13, and ADP-ribosyl cyclase are the most highly reactive tegument targets. The short alimentary tract proteins (MEGs, saposins) have the lowest correlation coefficient (r = 0.52), but almost all are more reactive with the hi-responder pool, by a factor of 2.21 times. However, the most reactive MEG-8.2 sits on the midline, while MEG-15 is an outlier, more reactive with the low-responder pool but at weak intensity (**Figure 4**).

### 3.6 There is a similarity in targets detected among IFNγR KO mice vaccinated with irradiated cercariae and rhesus macaques self-curing from *S. mansoni* and *S. japonicum*

**Figure 5:**
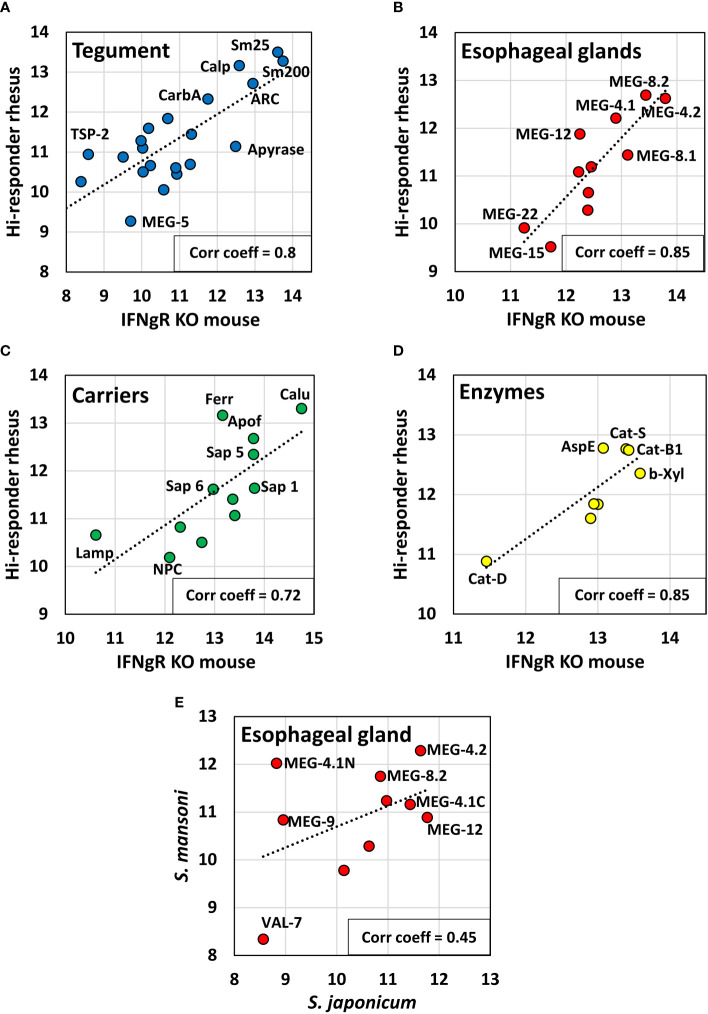
Scatter plots comparing the reactivity of hi-responder rhesus macaque sera with previously published data from IFNγR KO mice (19, Farias et al., 2021), screened on the same arrays, based on data in **Supplementary Table S3**, Sheet 3. The proteins were segregated by tissue of origin and function into: **(A)** tegument; **(B)** esophageal glands; **(C)** carriers; **(D)** enzymes. A strong relationship between the two data sets was revealed using Pearson’s correlation coefficient, r. **(E)** shows the relationship between esophageal gland proteins from hi-responder macaques and previously published data from rhesus macaques recovering from *S. japonicum* (18, Li et al., 2020), presented in **Supplementary Table S4**. The correlation r, between the two data sets was weaker.

Using all positive cell values, we compared the reactivity of the hi-responder macaques, which had eliminated most of their adult worms at 18 weeks, with already published data on the reactivity of the IFNγR KO mice (**Figure 5**). There was a good degree of concordance across the four arrays, with Pearson correlation coefficients between the two data sets of tegument (0.8), esophageal gland (0.85), carriers (0.72), and gut enzymes (0.85) (**Figures 5A–D**). It is apparent that the proteins highly reactive in the macaques self-curing from *S. mansoni* are similar to those eliciting a strong response in IFNγR KO mice. Assuming that the commonalities in macaque and mouse hosts represent potential protein targets of antibody-mediated protection, then for the tegument, Sm25, calpain, Sm200, ADP-ribosyl cyclase, and carbonic anhydrase are the most important. For the esophageal glands, the targets are the MEGs 4.1, 4.2, 8.1, 8.2, and 12. For the gastrodermal carriers, calumenin, ferritin heavy chain, apoferritin, and three saposins, and for the enzymes, cathepsins S and B1, asparaginyl endopeptidase, and beta xylosidase are the most shared. These data sets also indicate that proteins like MEGs 5, 15 and 22, Lamp, NPC-2, and cathepsin D show overall poor reactivity (**Figure 5**). Fortuitously, the hi-responder pool (Cy5) was reacted with short and long alimentary tract arrays previously used with the IFNγR KO mouse serum (Cy3), so a direct visual comparison can be made (**Supplementary Figure S2**). Although adjustment of the Cy3/Cy5 color balance needs care, visual inspection reveals that many identical runs of peptides are recognized by both sera. Good examples are the N-terminus of MEG-12 on the Array 1 and the C-terminus of VAL-7 on Array 2, both with “yellow” cells. However, other regions have discrete reactivity or greater intensity with one of the two sera. The N-terminus of MEG-4.1 on Array 1 is a good example (columns 41 and 42), where the reactive cells in descending order are three with mouse, four shared, and eight with rhesus macaque. On Array 2 (columns 21 and 22), there is a central region of cathepsin S with reactive cells: two mouse, one shared, five rhesus macaque, and two mouse. The previously mentioned poor reactivity of MEG-15 on Array 1 (columns 33–38) and cathepsin D on Array 2 (columns 27–34) is also very evident.

We were also able to compare the reactivity of the hi-responder macaques with an esophageal gland data subset, previously published (18) for macaques self-curing from *S. japonicum* (**Figure 5E**). The concordance between the two macaque groups responding to different schistosome species was lower, with a correlation coefficient of 0.45. MEG-12 was clearly more reactive in animals exposed to *S. japonicum*. The discrepancies are illustrated in a heatmap (**Supplementary Figure S3**) and in **Supplementary Table S4**. However, we highlight three regions within MEG-8.1, 8.2, and 8.3 proteins that had high sequence identity (>80%) between S. mansoni and S. japonicum and were strongly recognized by both macaque sera (labeled as regions 3, 4, and 5 in **Supplementary Figure S3**). Additionally, regions 1 and 2 with moderate sequence identity (40%–50%), in MEG-4.1 and 4.2, were strongly recognized by both macaque sera (**Supplementary Figure S3**). Our analysis suggests that these regions may exhibit a similar degree of spatial exposure in the native proteins of the two schistosome species. Although the N-terminus of MEG-12 (region 6) showed low sequence identity (~26%) in the two schistosomes, it was also strongly recognized by both macaque sera, again suggesting that these N-termini must be exposed and accessible to B-cell antigen receptors.

### 3.7 Specific epitope targets of rhesus macaque serum in array proteins

The most reactive regions in the proteins printed on the four arrays are presented as putative epitopes, together with their aggregate scores, in **Table 1** and **Supplementary Table S5**. This is equivalent to treating each replicate pair of cells on the array as a mini-ELISA, and then adding the adjacent cells to provide the score. In general, these scores are higher for the hi- than the lo-responder pool. On Array 1, short alimentary tract, peptide (P) 3 of MEG-8.2 stands out as more reactive with the lo-responder pool, but the rest are either equivalent or mostly much less reactive with the lo-responder serum. The most reactive peptides with scores >90,000 are P2 of saposin 5, P1 and P2 of MEG-4.2, P1 of MEG-8.2, P1 at the N-terminus of MEG-4.1, and P1 on MEG-12. On Array 2, long alimentary tract, all peptides with scores >75,000 are most reactive with the hi-responder serum, the most notable being P2 and P3 of the ferritin heavy chain, P4 of asparaginyl endopeptidase, P1 and P4 of cathepsin S, and P2 of cathepsin B1.2. Among the short tegument proteins (Array 3), only three stand out, the remainder having aggregate scores of<40,000. These are the very strongly reacting P1, P2, and P3 of Sm25; P1 of ADP-ribosyl cyclase; and the N-terminal P1 of Sm13. The long tegument proteins on Array 4 were weakly reactive, with only calpain and Sm200 surface protein having scores >30,000. It is notable that while P2–P5 of calpain reacted with the hi-responder pool, P2, P4, and P5 also reacted with the lo-responder pool. The same is true of P5 and P6 of Sm200, with lo responder scores very similar to hi responders. This appears to argue that host IgG responses against these long tegument proteins are not associated with protection in this model.

**Table 1:** Principal reactive epitopes.

Tegument	P#	Epitope
Sm13	1	EPEPEPEPVPVSRNS
Sm25	3	PDGFPEYEFLNETSI
Sm25	2	QEAFHRNSDPDGFPE
Sm25	1	SNSIITDEDYDHYNS
ADP Ribosyl cyclase	1	NISCSEIWNSFESIL
Esophageal gland	P#	Epitope
MEG-4.1	1	SPLDDRFNDVNTINK
MEG-4.1	2	INKKQFTEEEFSRLI
MEG-4.2	1	DIEPRIQKEYYYNLH
MEG-4.2	2	RIQKEYYYNLHENNS
MEG-8.1	1	FFDLFSEQEFHPINH
MEG-8.2	3	SMFGSSDSSSGTNNK
MEG-8.2	1	VSKPTATVKPQPVNK
MEG-12	1	SGENYEQQLQQPKAY
VAL-7	1	PYDPIYPEDPYYLPG
Gastrodermis	P#	Epitope
Asparaginyl	4	TESSYGTFCDDPTIT
endopeptidase	1	YDDIAYNLMNPFPGK
Cathepsin B1.2	2	SKENHTGCEPYPFPK
Cathepsin B1.2	3	KCEHHTKGKYPPCGS

The amino acids in red indicate the reactivity center of the epitope.

## 4 Discussion

We first consider the choice of proteins for printing on the four arrays. The axiom for selection was that proteins involved in protective immunity must be exposed on or secreted from external surfaces of the intra-mammalian parasite to be accessible to immune effector mechanisms. Internal proteins may be highly immunogenic but that is of no value if, in the live parasite, they are inaccessible to immune effectors. The proteins analyzed in this study were selected based on our extensive proteomic and transcriptomic analyses (listed in Farias et al., 2021 (19)). These studies were underpinned by bioinformatic analysis of transcripts, largely by the late Dr. Ricardo DeMarco. Localization of gene expression was performed by whole mount *in situ* hybridization (WISH) (20) and of protein by immunocytochemistry and high-resolution confocal microscopy, using monospecific antibodies (for the tegument, see (21)).

The arrays were designed and experiments executed before publication of the results of single-cell sequencing from disaggregated adult worms (22) and the creation of SchistoCyte atlas, a database searchable for the pattern of expression of schistosome gene transcripts by tissue (23). The database has already been interrogated using the tegument proteins on Arrays 3 and 4 (21); all were found in one or more of the eight tegument clusters, apart from LMWP (potentially parenchymal), which has been removed from the molecular model of the tegument apical surface. The alimentary tract proteins have now similarly been searched against SchistoCyte (**Supplementary Table S6**). Eight of the nine esophageal gland MEG proteins and VAL-7 are confirmed as exclusively expressed in the esophageal gland cluster. The exception is MEG-12, which was present diffusely at very low levels throughout. However, *in situ* hybridization has revealed that MEG-12 is expressed in the small anterior esophageal gland (24), which is not mentioned by Wendt et al. (22), so this tissue may not have been captured by their study. All six saposins, asparaginyl endopeptidase, and several of the cathepsins are located exclusively in the gastrodermis (gut cluster). Other enzymes, plus NPC-2 and LAMP (lysosomal-associated membrane protein) are present not only in the gastrodermis but also in other tissues. A recent comparative proteomic profile of microdissected male esophageal gland (ESO) versus extreme body posterior (BE) revealed that some proteins previously thought exclusive to the gastrodermal compartment do function in other worm tissues (25). Apoferritin may fall into this category since SchistoCyte atlas locates it not only in the gastrodermis but also in the internal parenchyma cluster/tissue. The Ferritin h chain is located in the tegument clusters, barely detected in the gut, and lacks a signal sequence (but then so does calpain from the tegument surface). SchistoCyte places calumenin in muscle cells, which lie beneath the tegument; indeed, it may derive from the sarcoplasmic reticulum of those cells. Thus, it is possible that the high reactivity scores of ferritin h chain and calumenin occur, since, due to their internal location, they had not been subject to selection pressure from the immune system in the live worm. Our stated axiom thus excludes them as vaccine candidates.

A clear message from the comparison of hi- versus lo-responder pools is that the dominant factor in the self-cure process is antibody titer, not a failure of lo responders to identify specific targets. The experimental animals were outbred, so diversity of MHC haplotypes might be expected to cause variation in antigen presentation. Using pools of two animals would have smoothed out some variation, but the single unique epitope detected by lo-responder animals in MEG-8.2 was the exception. This argues for a universality of protein epitopes mediating protection. The arrayed proteins were approximately twice as reactive with the hi-responder pool, the exceptions being five of the long tegument proteins (Sm200, calpain, two annexins and alkaline phosphatase). Does this mean that they are not involved in protection in the self-cure model? Another important conclusion is that, except for Sm25, tegument proteins were significantly less reactive than alimentary tract proteins (despite being screened with a higher antibody concentration). This could simply be a function of the degree of immunostimulation. Very probably, the release of alimentary tract proteins into the bloodstream is much greater than the release of tegument membranocalyx, so the former are more likely to stimulate antibody production. Worms *in vivo* do not bind leukocytes (26), and we have argued that exposed tegument proteins must have been under selection pressure for “immunological silencing” (27).

The tegument exception, Sm25 (annotated as Smp_346910 on WormBase Parasite) deserves more detailed scrutiny. It was characterized before the genomic era as a tegument glycoprotein (28) and localized by immunocytochemistry (29) but does not appear to have been tested for protective potential. Direct evidence for its surface accessibility comes from proteomic analysis of culture supernatants from trypsinized live worms, where it was identified among a select group of host and worm proteins. The host proteins included immunoglobulins, complement factors, and CD44, clearly in the most external location. The other worm proteins were calpain, Sm200, three annexins, and two endophilins. Our best guess is that this group of proteins is involved in membranocalyx secretion deep at the base of tegument pits (21). They are accessible to trypsin (Mr ~ 20kDa) in the live worm. Would Sm25 be accessible to IgG (Mr ~ 150,000 kDa)? The protective potential of calpain (5) argues that it would.

Esophageal gland products were also preferentially detected by the hi-responder pool, namely, MEGs 4.1, 4.2, 8.1, 8.2, and 12. They are released into the anterior and posterior compartments of the esophageal lumen where initial processing of ingested blood takes place (20). Their precise roles in the uncoating of erythrocytes and disabling of leukocytes has not been established. However, co-detection of parasite protein and host IgG has been reported for MEGs 4.1, 4.2, 8.2, 9, 11, and VAL-7 in the esophageal lumen of *S. japonicum* worms from rhesus macaques (12). Additionally in the context of MEG-12 secreted from the anterior gland (24), there is strong morphological evidence in *S. japonicum* for blocking (constipation) of the secretory process. There is also much IgG deposition on the luminal surface, leading to a build-up of giant vesicles in the esophageal lining tissues (12). If this could be ascribed to antibody directed against the strongly reactive N-terminus of MEG-12, it would be an ideal vaccine candidate. A group of gastrodermal proteases are also more strongly recognized by the hi-responder pool. Antibody blocking of their activity by immune complex formation or neutralization is an attractive way to starve the worms of nutrients. Indeed, cathepsin B1 has been put forward as a vaccine candidate (30). A potential downside is the strong acidic environment in the gut lumen; the effect of a low pH on antibody activity does not appear to have been tested. Among the proteases, asparaginyl endopeptidase may be an attractive target, since it activates the other cathepsins in the hemoglobin hydrolysis cascade (31). Its neutralization could thus block the cascade.

The similarities in reactivity between the hi responder and IFNγR KO mouse sera reinforce the identities of the proteins mediating protection in the two models. For the tegument, Sm25 stands out, with Sm13, ADP ribosyl cyclase, and carbonic anhydrase as weaker but positive targets. Among esophageal gland products, the MEG-4 and 8 families are the most reactive, while MEG-12 is seen more strongly by the hi-responder pool. For the carriers, calumenin and ferritin are strongly detected by both sera but appear to be excluded by SchistoCyte. Apoferritin may be worth consideration, and saposin 5 is strongly detected by both sera. The problem with saposins as vaccine targets is that their multiplicity suggests a considerable overlap in uptake capabilities. We surmise a need to target all of them to achieve a biological effect, and they have for the moment been set aside. Asparaginyl endopeptidase and cathepsins B1 and S are prominent in both sera. The much lower correlation in the reactivities of rhesus macaques undergoing self-cure of *S. mansoni* and *S. japonicum* infections is striking. This could reflect the evolutionary distance, variously estimated as between 10 and 70 million years, between “basal” *S. japonicum* and *S. mansoni*, very much a parasite of *Homo sapiens* emerging in the last 0.3–0.4 million years (32). MEG-12, the N-terminus of MEG-4.1, and MEG-8.3 are the proteins with the stronger reaction in *S. japonicum*.

The foregoing appraisals are based on aggregate scores for the entire printed protein. A particular advantage of the peptide array approach is that it facilitates analysis at the level of a single antigenic epitope. The superimposed images of reactions to the two alimentary tract arrays highlight not only the existence of reactivities unique to macaque or mouse but also regions of overlap that may represent common epitopes, or even universal epitopes. As observed here and in unpublished work from other host species, the two N-terminal regions of Sm25 appear to be in this last category. Detailed analysis can also highlight “hot spots” within an otherwise poorly reactive subject. A region adjacent to the C-terminus of VAL-7 comes into this category in the IFNγR KO mouse.

How can we use the information generated in this study? It seems unlikely, but not impossible, that a single “magic bullet” antigen administered as a vaccine could replicate features of self-cure. Given the reported characteristics of the process (11–13), we have argued that sustained immunological pressure against multiple targets is needed over an extended period to eliminate established adults or challenge larvae in a self-cured macaque. This cannot be the whole explanation because self-cure proceeds at widely different rates in individual macaques (t^1/2^ 2.8–8.7 weeks; 13). This is equally true of the elimination of a challenge of self-cured animals where fast-responder animals showed virtually complete immunity, whereas in slow-responders, blood feeding, judged by circulating antigen (CAA) levels, started before the infection was controlled but not completely eliminated. Proteomic and metabolomic analyses of rhesus plasma over this prolonged time course should provide pointers to the missing dimensions in the self-cure process, additional to the identity of target antigens.

In a proof of principle experiment, we have shown the feasibility of joining several short epitopes in a single construct that will elicit multiple antibody specificities in experimental animals (18). This is the approach we now propose to take with the principal reactive regions of tegument and alimentary tract proteins that we have identified. We will combine them in a single synthetic construct to assess their protective capacity in animal models and fine tune the composition of the construct to optimize its vaccine potential.

## Data availability statement

The original contributions presented in the study are included in the article/**Supplementary Material**, further inquiries can be directed to the corresponding authors.

## Ethics statement

The animal study was approved by Institutional Animal Care and Use Committee at Biomedical Primate Research Center, Rijswijk, The Netherlands, and by the Biology Department Ethics Committee, University of York, UK. The study was conducted in accordance with the local legislation and institutional requirements.

## Author contributions

RW: Conceptualization, Data curation, Formal Analysis, Project administration, Supervision, Writing – original draft. GV: Formal Analysis, Investigation, Methodology, Writing – original draft. MK: Data curation, Methodology, Visualization, Investigation, Writing – original draft. AN: Methodology, Software, Visualization, Writing – review & editing. SJ: Investigation, Methodology, Resources, Writing – review & editing. LL: Conceptualization, Funding acquisition, Resources, Writing – review & editing. LF: Conceptualization, Data curation, Formal Analysis, Funding acquisition, Investigation, Methodology, Project administration, Resources, Writing – original draft.
