# Supplementary material for: Antigenic epitope targets of rhesus macaques self-curing from Schistosoma mansoni infection
Source: Front Immunol. 2024 Feb 23;14:1269336. doi: 10.3389/fimmu.2023.1269336 (PMC10921417; doi:10.3389/fimmu.2023.1269336)
Supplement: Supplementary file 1 [file Presentation_1.pptx]

## Slide 1
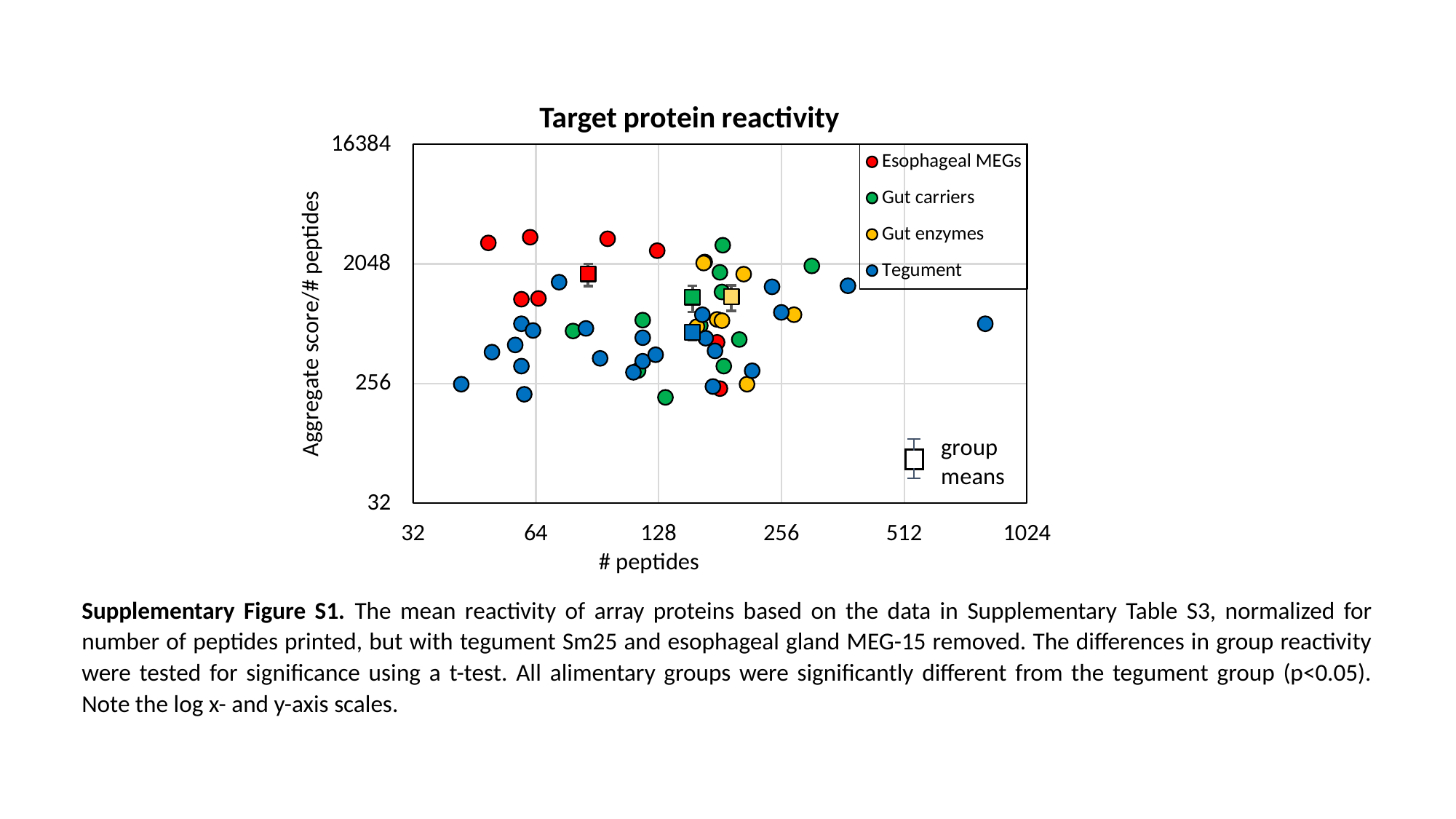

Supplementary Figure S1. The mean reactivity of array proteins based on the data in Supplementary Table S3, normalized for number of peptides printed, but with tegument Sm25 and esophageal gland MEG-15 removed. The differences in group reactivity were tested for significance using a t-test. All alimentary groups were significantly different from the tegument group (p<0.05). Note the log x- and y-axis scales.
