# Supplementary material for: Antigenic epitope targets of rhesus macaques self-curing from Schistosoma mansoni infection
Source: Front Immunol. 2024 Feb 23;14:1269336. doi: 10.3389/fimmu.2023.1269336 (PMC10921417; doi:10.3389/fimmu.2023.1269336)
Supplement: Supplementary file 2 [file Presentation_2.pptx]

## Slide 1
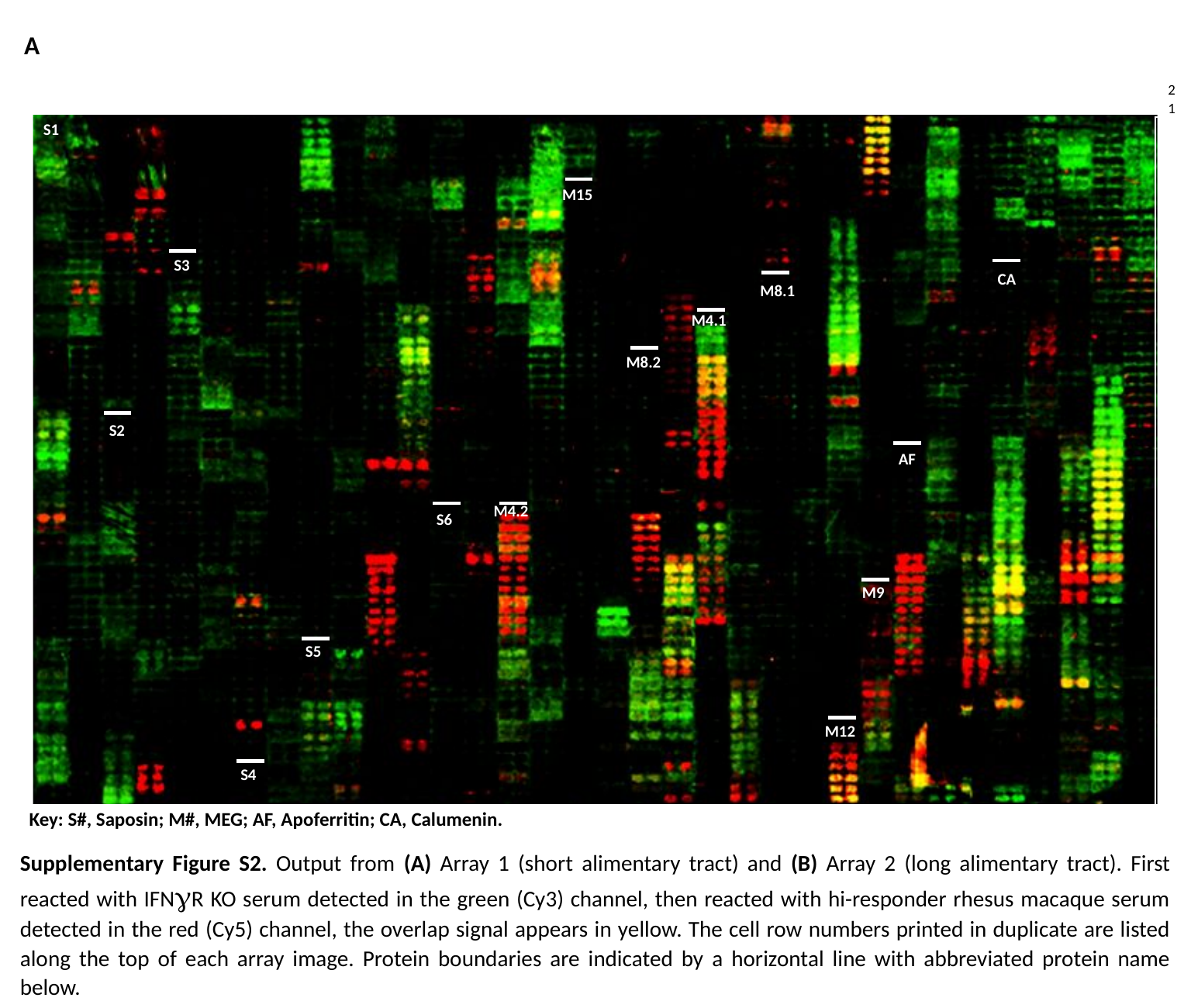

A
| 2 | 4 | 6 | 8 | 10 | 12 | 14 | 16 | 18 | 20 | 22 | 24 | 26 | 28 | 30 | 32 | 34 | 36 | 38 | 40 | 42 | 44 | 46 | 48 | 50 | 52 | 54 | 56 | 58 | 60 | 62 | 64 | 66 | 68 |
| --- | --- | --- | --- | --- | --- | --- | --- | --- | --- | --- | --- | --- | --- | --- | --- | --- | --- | --- | --- | --- | --- | --- | --- | --- | --- | --- | --- | --- | --- | --- | --- | --- | --- |
| 1 | 3 | 5 | 7 | 9 | 11 | 13 | 15 | 17 | 19 | 21 | 23 | 25 | 27 | 29 | 31 | 33 | 35 | 37 | 39 | 41 | 43 | 45 | 47 | 49 | 51 | 53 | 55 | 57 | 59 | 61 | 63 | 65 | 67 |
S1
M15
S3
CA
M8.1
M4.1
M8.2
S2
AF
M4.2
S6
M9
S5
M12
S4
| | | | | | | | | | | | | | | | | | | | | | | | | | | | | | | | | | |
| --- | --- | --- | --- | --- | --- | --- | --- | --- | --- | --- | --- | --- | --- | --- | --- | --- | --- | --- | --- | --- | --- | --- | --- | --- | --- | --- | --- | --- | --- | --- | --- | --- | --- |
Key: S#, Saposin; M#, MEG; AF, Apoferritin; CA, Calumenin.
Supplementary Figure S2. Output from (A) Array 1 (short alimentary tract) and (B) Array 2 (long alimentary tract). First reacted with IFNγR KO serum detected in the green (Cy3) channel, then reacted with hi-responder rhesus macaque serum detected in the red (Cy5) channel, the overlap signal appears in yellow. The cell row numbers printed in duplicate are listed along the top of each array image. Protein boundaries are indicated by a horizontal line with abbreviated protein name below.

## Slide 2
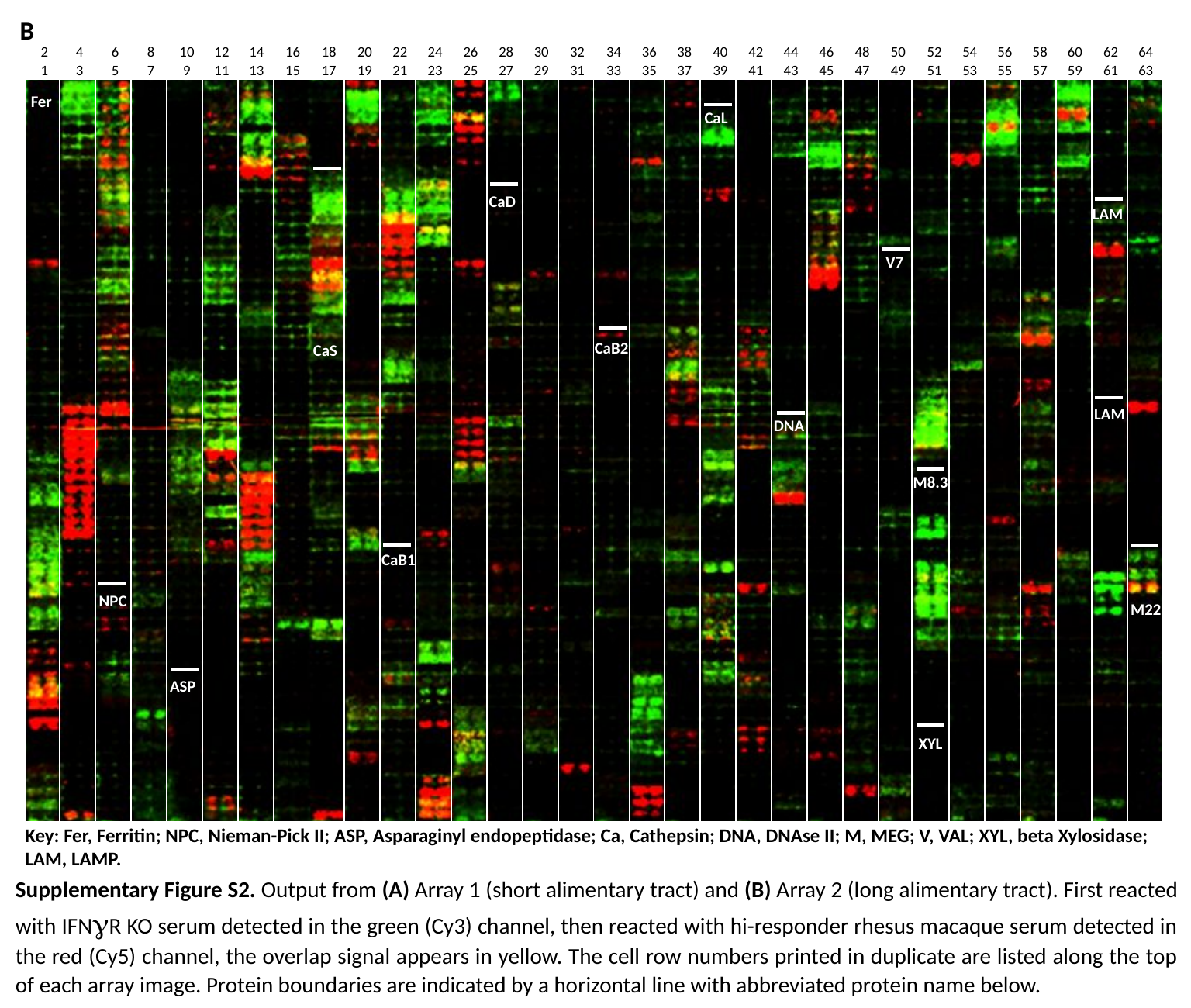

B
| 2 | 4 | 6 | 8 | 10 | 12 | 14 | 16 | 18 | 20 | 22 | 24 | 26 | 28 | 30 | 32 | 34 | 36 | 38 | 40 | 42 | 44 | 46 | 48 | 50 | 52 | 54 | 56 | 58 | 60 | 62 | 64 |
| --- | --- | --- | --- | --- | --- | --- | --- | --- | --- | --- | --- | --- | --- | --- | --- | --- | --- | --- | --- | --- | --- | --- | --- | --- | --- | --- | --- | --- | --- | --- | --- |
| 1 | 3 | 5 | 7 | 9 | 11 | 13 | 15 | 17 | 19 | 21 | 23 | 25 | 27 | 29 | 31 | 33 | 35 | 37 | 39 | 41 | 43 | 45 | 47 | 49 | 51 | 53 | 55 | 57 | 59 | 61 | 63 |
| | | | | | | | | | | | | | | | | | | | | | | | | | | | | | | | |
| --- | --- | --- | --- | --- | --- | --- | --- | --- | --- | --- | --- | --- | --- | --- | --- | --- | --- | --- | --- | --- | --- | --- | --- | --- | --- | --- | --- | --- | --- | --- | --- |
Fer
CaL
CaD
LAM
V7
CaB2
CaS
LAM
DNA
M8.3
CaB1
NPC
M22
ASP
XYL
Key: Fer, Ferritin; NPC, Nieman-Pick II; ASP, Asparaginyl endopeptidase; Ca, Cathepsin; DNA, DNAse II; M, MEG; V, VAL; XYL, beta Xylosidase; LAM, LAMP.
Supplementary Figure S2. Output from (A) Array 1 (short alimentary tract) and (B) Array 2 (long alimentary tract). First reacted with IFNγR KO serum detected in the green (Cy3) channel, then reacted with hi-responder rhesus macaque serum detected in the red (Cy5) channel, the overlap signal appears in yellow. The cell row numbers printed in duplicate are listed along the top of each array image. Protein boundaries are indicated by a horizontal line with abbreviated protein name below.
