# Supplementary material for: Antigenic epitope targets of rhesus macaques self-curing from Schistosoma mansoni infection
Source: Front Immunol. 2024 Feb 23;14:1269336. doi: 10.3389/fimmu.2023.1269336 (PMC10921417; doi:10.3389/fimmu.2023.1269336)
Supplement: Supplementary file 3 [file Presentation_3.pptx]

## Slide 1
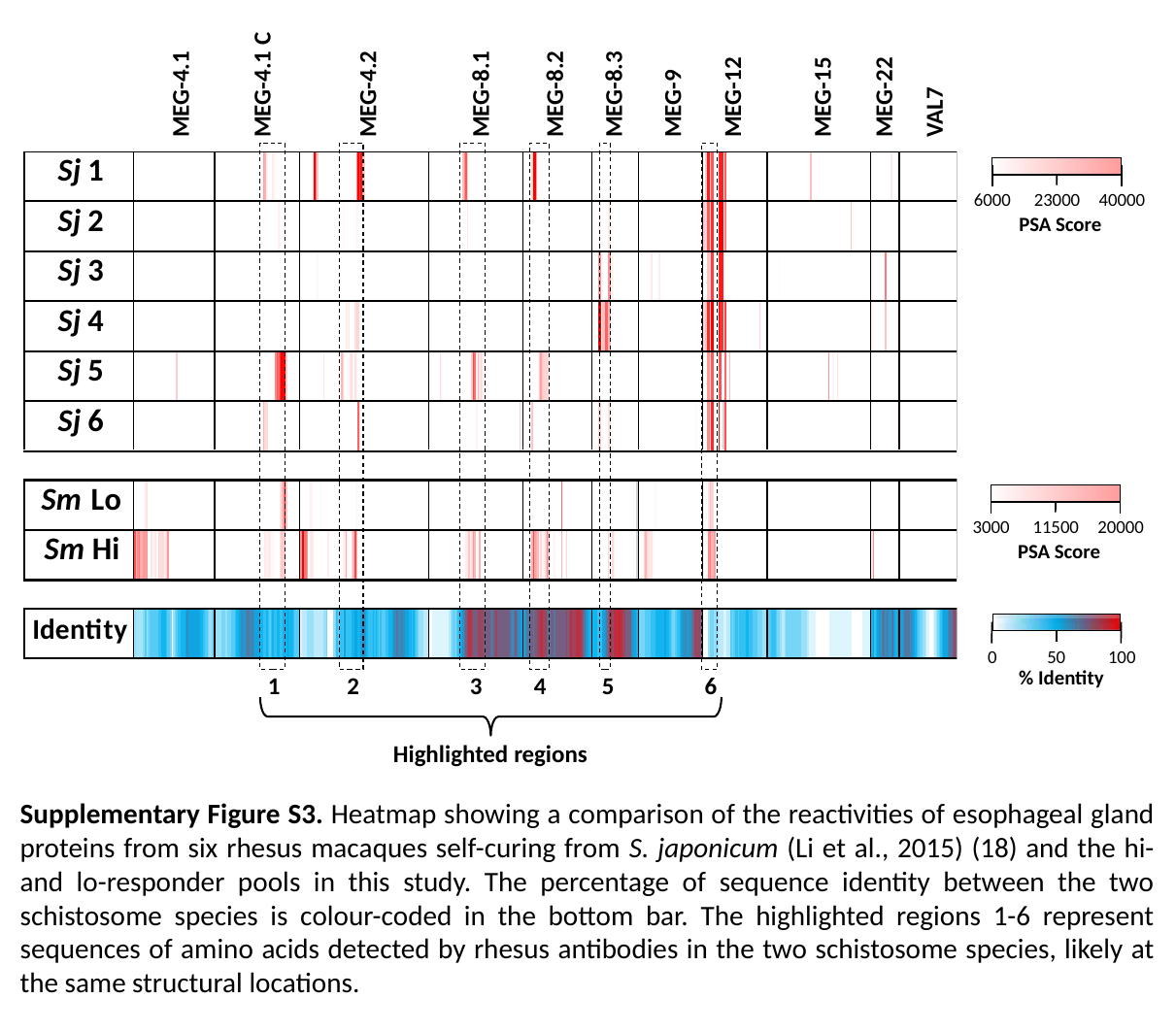

MEG-4.1
MEG-4.2
MEG-8.1
MEG-8.2
MEG-8.3
MEG-9
MEG-12
MEG-15
MEG-22
VAL7
MEG-4.1 C
6000
23000
40000
PSA Score
11500
20000
3000
PSA Score
0
50
100
% Identity
6
1
2
3
4
5
Highlighted regions
Supplementary Figure S3. Heatmap showing a comparison of the reactivities of esophageal gland proteins from six rhesus macaques self-curing from S. japonicum (Li et al., 2015) (18) and the hi- and lo-responder pools in this study. The percentage of sequence identity between the two schistosome species is colour-coded in the bottom bar. The highlighted regions 1-6 represent sequences of amino acids detected by rhesus antibodies in the two schistosome species, likely at the same structural locations.
